# Confluence of Plant-Based Dietary Patterns and Polygenic Risk for Venous Thromboembolism

**DOI:** 10.1016/j.jacadv.2024.101321

**Published:** 2024-10-16

**Authors:** Nikolaos Tsaftaridis

**Affiliations:** Anticoagulation and Clinical Thrombosis Services, Institute of Health Systems Science, Feinstein Institutes of Medical Research, Northwell Health, Manhasset, New York, USA

**Keywords:** nutritional epidemiology, plant-based diet, polygenic risk score, thrombosis, VTE risk

**Figure:**
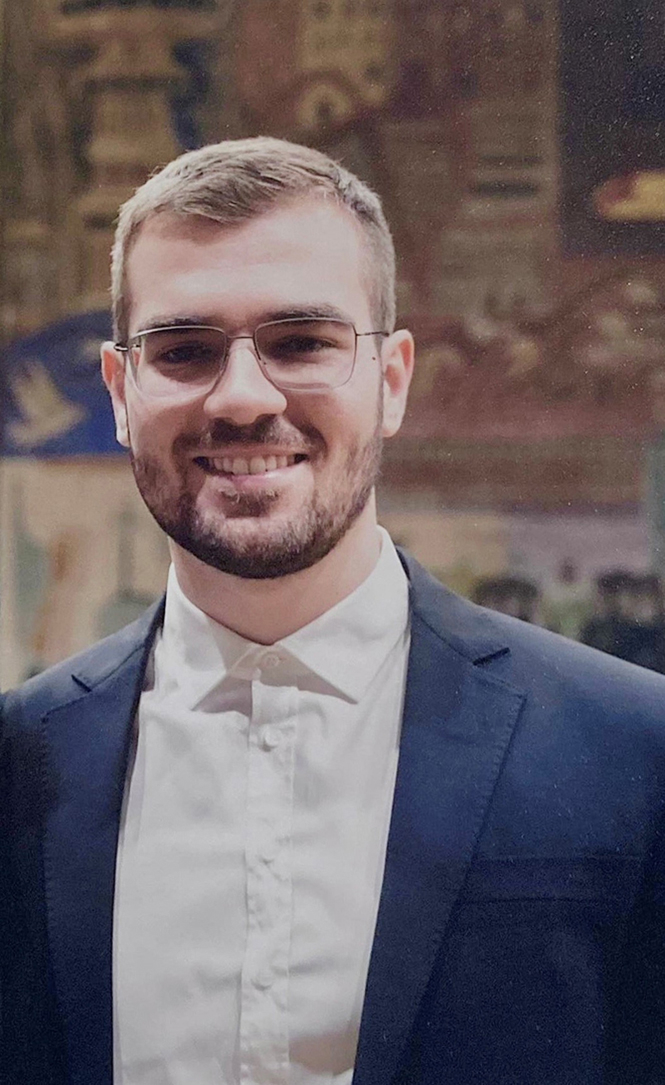

The spectrum of venous thromboembolic (VTE) risk factors has not yet been fully characterized. While a high body mass index is a probable risk factor for VTE,^1^^,^^2^ the contributions of lifestyle choices including specific eating patterns to disease risk have been disputed, in contrast to what is true for other cardiovascular outcomes, such as myocardial infarction.^3^^,^^4^

Polygenic risk scores (PRS) consisting of differing numbers of single-nucleotide polymorphisms have been proposed as having potential for identifying genetic profiles conferring increased VTE risk, sometimes on par with individual clinical risk factors.^5^^,^^6^ However, there is a relative paucity of evidence around their clinical utility^5^ and it is not known whether the proposed VTE-associated genotypes would influence risk through lifestyle choices or disease-specific mechanisms. These are actionable questions relevant to risk assessment, diagnostic workup, and prevention strategies. Furthermore, if the 2 proposed risk factors are independent, there may be a need for more advanced genetic risk assessment modalities to personalize prevention than are currently available.

The study by Guo et al^7^ in this issue of *JACC: Advances* investigates the potential independent and confluent effects of a plant based dietary pattern and a VTE-specific polygenic profile on disease incidence in populations of European ancestry.^7^ The longitudinal data used for the retrospective analysis include nutritional habit questionnaires and a PRS that has been associated with increased VTE incidence in the White ancestry population.^8^

In the context of a nutritional epidemiology approach, the authors utilize an observational retrospective design to assess the chronic cumulative effects of nutritional patterns on the cardiovascular outcome of interest. Given the hypothesized dispersion of risk across the cohort and concomitant long exposures necessary for an event, this question would be difficult to study outside an observational framework with long follow-up, such as the design of this study.

According to the new findings, higher plant food consumption reflected in a high plant-based diet index score is associated with decreased incidence of VTE and its constituents deep vein thrombosis and pulmonary embolism. Similar were the findings for a lower PRS. The HR for VTE incidence comparing populations with an elevated PRS to those with a baseline level PRS aligns with findings reported in prior literature.^6^ Per the authors, these 2 factors (high plant-based diet index and PRS) seem to not be strongly interacting.^7^ Common risk factors such as male gender, higher body mass index, unfavorable social determinants of health and low levels of physical activity, smoking and history of cancer or cardiovascular disease were revealed to be more common in patients who developed VTE, as expected. Additionally, the genetic risk seemed to be more pronounced in younger and male patients.

This paper has parallels with 2 previous studies using data from the same database to research VTE risk, with congruent results. The first investigated whether the same PRS predicts VTE in cancer patients from the UK Biobank.^9^ The second study, by Zhang et al,^10^ is even more similar, having examined the interaction and contributions of a broadly defined “healthy lifestyle” and genetic predisposition in relation to VTE.

Even though this study design is not readily able to establish causative relationships, given the methodological constraints posed by the nature of the scientific question it offers valuable insight. Exact predetermined criteria on when epidemiological data can be trusted to establish causality are currently the subject of debate.^11^^,^^12^

Other potential considerations include that there is no established standard definition of a plant-based diet,^13^ and using nutritional recall questionnaires is notoriously inaccurate.^14^ However, the authors provide detailed reporting of what they consider a healthful, plant-based diet, and since we are mainly concerned with overall dietary patterns and not specific nutrients or calorie counts, the recall inaccuracy may be less impactful. Readers are left to determine for themselves whether they agree with the authors’ choices on which foods are considered healthful and which are not.

One thing that is not clear is to what extent the participant sample from the UK Biobank data used to derive the PRS overlaps the participant sample for this study’s analysis.^8^ A similar limitation is acknowledged by the authors of the aforementioned study by Guman, et al.^9^ If there is significant overlap, the ability of the PRS to predict VTE incidence has been overestimated in the current study. For the PRS to be established as reflective of actual VTE risk, it needs to be validated in external populations.^15^

With this potential caveat, the study significantly contributes to the discussion around nutritional risk factors for VTE and the potential utility of a broader genetic risk assessment for VTE. Well-designed prospective cohort studies need to be undertaken to establish more confidence in the findings prior to drawing conclusions for clinical practice.

## Funding support and author disclosures

The author has reported that they have no relationships relevant to the contents of this paper to disclose.
